# Effects of Blood Flow Restriction Training on Strength and Functionality in People With Knee Arthropathies: A Systematic Review and Dose-Response Meta-Analysis of Randomized Controlled Trials

**DOI:** 10.1155/tsm2/3663009

**Published:** 2025-04-10

**Authors:** Daniel C. Ogrezeanu, Luís Suso-Martí, Rubén López-Bueno, Pedro Gargallo, Rodrigo Núñez-Cortés, Carlos Cruz-Montecinos, Lars Louis Andersen, José Casaña, Nicholas Rolnick, Joaquín Calatayud

**Affiliations:** ^1^Exercise Intervention for Health Research Group (EXINH-RG), Department of Physiotherapy, University of Valencia, Valencia, Spain; ^2^National Research Centre for the Working Environment, Copenhagen, Denmark; ^3^Department of Physical Medicine and Nursing, University of Zaragoza, Zaragoza, Spain; ^4^Department of Physiotherapy, Faculty of Medicine and Health Science, Catholic University of Valencia, Valencia, Spain; ^5^Physiotherapy in Motion Multispeciality Research Group (PTinMOTION), Department of Physiotherapy, University of Valencia, Valencia, Spain; ^6^Department of Physical Therapy, Faculty of Medicine, University of Chile, Santiago, Chile; ^7^The Human Performance Mechanic, New York, New York, USA

**Keywords:** BFR, knee osteoarthritis, quadriceps strengthening, rheumatoid arthritis, strength training, vascular occlusion

## Abstract

**Background:** Previous meta‐analyses show contrasting findings regarding the effects of blood flow restriction training (BFRT) in different knee conditions. Furthermore, no previous dose-response analysis has been conducted to determine the dose of BFRT required for maximal strength and functionality adaptations.

**Objective:** To analyze the evidence on the effects of BFRT on strength and functionality in patients with knee osteoarthritis or rheumatoid arthritis through a systematic review with dose-response meta‐analysis.

**Methods:** Included studies met the following criteria: participants with knee osteoarthritis or rheumatoid arthritis; low-load resistance BFRT as intervention; control group with traditional moderate or high intensity resistance training (MIRT and HIRT); include muscle strength and functionality as primary and secondary outcome measures, respectively; and only randomized controlled trials. A random-effects and a dose-response model estimated strength and functionality using estimates of the total repetitions performed.

**Results:** We included five studies with a sample of 205 participants. No statistically significant differences were found between BFRT and MIRT or HIRT for strength (SMD = −0.06; 95% CI = −0.78–0.67; and *p* > 0.05) and functionality (SMD = 0.07; 95% CI = −0.23–0.37; and *p* > 0.05). We found an inverted U-shaped association between the increase in total repetitions and strength gain and between the increase in total repetitions and functional improvement.

**Conclusions:** People with knee osteoarthritis or rheumatoid arthritis can use low-load BFRT for strength and functionality as a similarly effective alternative to MIRT and HIRT. A total of 2000 repetitions per BFRT program are necessary to maximize strength gains in these patients, while functional improvement requires 1800 total repetitions.

## 1. Introduction

Osteoarthritis (OA) and rheumatoid arthritis (RA) are examples of chronic arthropathies. OA is a chronic progressive multifactorial joint disease that can affect the many tissues of the joint [1]. Knee OA is one of the leading causes of global functional disability health burden among adults [2, 3]. Chronic pain and stiffness are its dominant symptoms [1], but knee extensor muscle weakness has been shown to be a risk factor for the development of symptomatic knee OA [4], a major independent contributor to falls [5], and increased activity limitations [6]. On the other hand, RA is a chronic, autoimmune and highly inflammatory disease that affects mainly large joints and extraarticular tissues, often leading to significant functional disability [7]. RA patients show a reduction of muscle mass and strength with stable or increased adiposity [8] that contributes to increased physical disability [9] and risk of comorbidities and mortality [10]. Thus, both OA and RA are associated with muscle weakness that may benefit from physical rehabilitation.

Current guidelines strongly recommend exercise for the management of OA and RA, although current evidence is insufficient to recommend specific exercise modalities [11, 12]. Traditional strength programs for impairment mitigation should be of sufficient intensity to stimulate anabolism and limit muscle loss. However, resistance training with moderate to high-intensity loads (60%–80% of 1 repetition maximum (1RM)) [13] could likely be a barrier to participation secondary to discomfort and pain from the physical and psychological symptomatology of RA and OA [14–19]. Thus, the challenge lies in implementing alternative exercise interventions that are effective in combating muscle weakness yet tolerable to encourage long-term adherence.

Low-load (20%–30% 1RM) resistance training with concurrent blood flow restriction (BFR) [20–22] has emerged as a training modality that could be tolerable to load compromised patients that provides similar benefits to high intensity exercise. BFR training (BFRT) is typically applied with a pressurized cuff on the proximal aspect of a limb to compress the underlying vasculature during exercise. In theory, the interaction between mechanical tension and metabolic stress (hypoxia and accumulation of metabolites, reactive hyperemia) leads to an enhanced intramuscular signaling that would explain the benefits seen with BFRT in other populations [23]. However, several previous systematic reviews and meta‐analyses have raised apparently contrasting findings about the effects of BFRT in different knee conditions [23–28]. While some of these reviews reported that BFRT appears to be a promising strategy for muscle strength gains [23, 24, 26], muscle mass gains [23, 24] or improving function [23, 24], others indicated that BFRT may not have greater efficacy compared with moderate to high-intensity resistance training for various clinical outcomes [25, 27, 28], which is still a positive finding as it signifies BFRT is roughly equivalent to moderate to high-intensity resistance training, and could still have its utility in load compromised individuals. While there was considerable overlap between the studies included across these reviews, it should be noted that their precise eligibility criteria varied, and so did the included knee conditions, or the intervention loads that were compared. A more recent meta-analysis further supports the potential of BFRT in the disease management of patients with OA and RA, yet it does so with a high degree of heterogeneity due to pooling dissimilar intervention loads and self-reported function or physical function outcomes [29]. In addition, previous reviews have not considered other crucial variables for exercise prescription such as the number of repetitions within a training program. Importantly, to date, no previous review has conducted a dose-response meta-analysis that could allow a better understanding of the dose of BFRT required to maximize strength and functionality adaptations. We aimed to analyze the evidence for the effects of BFRT on strength and functionality in individuals with a diagnosis of knee OA or RA through a systematic review and dose-response meta‐analysis. The secondary objective was to analyze the quality of the evidence.

## 2. Materials and Methods

### 2.1. Protocol Design

The search and selection of articles adhered to PRISMA-P guidelines [30] and were conducted after registering the protocol with PROSPERO platform (CRD42023401715).

### 2.2. Study Selection and Eligibility Criteria

Two authors (D.C.O. and P.G.) applied the same criteria to identify and select the included articles based on the PICOS criteria [31]. To match the inclusion criteria, a study had to include participants diagnosed with knee OA or RA (P); have traditional BFRT (constant BFR, applied throughout all the sets and interset rests of an exercise uninterruptedly) with low-load resistance (20%–40% 1RM) as the intervention (I); have a control group with conventional moderate intensity resistance training (MIRT) or high intensity resistance training (HIRT) (60%–80% 1RM) (C); include muscle strength as a primary outcome measure or physical function as secondary (O); and the study type should be a randomized controlled trial (S). Also, the articles should be written in English or Spanish. The search was conducted from database inception until 30 June 2024.

### 2.3. Data Sources and Search Strategy

The search was conducted using the following electronic databases: PubMed, Ebsco, Cochrane Library, and PEDro. Due to concerns about the quality of gray literature, only peer-reviewed sources were included to ensure a higher level of rigor and reliability. In addition, reference lists were manually searched to identify studies that were not found through database searching.

The search terms included the following keywords and their possible variants, synonyms, or combinations: “blood flow restriction,” “therapy,” “training,” “exercise,” “KAATSU,” “osteoarthritis,” and “rheumatoid arthritis”. The keywords were the same in all the databases, yet some adjustments were required to match each database's particularities, as can be seen in Supporting File 1.

### 2.4. Data Extraction

Data extraction was performed by two authors (D.C.O. and P.G.). The results were compared, and any disagreements/discrepancies were resolved by a third and fourth reviewers (J.C. and L.S.M.). The information extracted from the studies regarded: 1) sample: sample size of each group (n) and characteristics of study participants (age, sex, type of pathology, and eligibility); 2) methodology: characteristics of the intervention protocol (duration, frequency, intensity, volume, types of exercises, rest times, form of occlusion application, pressure used, and cuff size); and 3) effects: type of outcome, result measurement, measurement instrument or scale, statistical data, results obtained for dynamic and isometric muscle strength, and physical function. After inclusion of the articles in our systematic review with meta-analysis (*n* = 5), data were extracted from each study. When available, data were extracted in the form of mean and standard deviation, mean difference or percentage of change, and sample size of the studies to perform the meta-analysis. When data were not in the expected format, we requested information from the respective authors. If the authors did not reply or if the data provided by them were not clear, means and SD from the figures provided in the articles were extrapolated using the WebPlotDigitizer (Version 4.6; Pacifica, California, USA) tool [32].

### 2.5. Assessment of Risk of Bias

The risk of bias of randomized trials was evaluated with the Risk of Bias 2 (RoB 2) tool by two independent authors (D.C.O. and P.G.) [33]. Similarly, any disagreements/discrepancies were resolved by a third and fourth reviewers (J.C. and L.S.M.). Five items were assessed, namely, randomization process, deviations from intended interventions, missing outcome data, measurement of the outcome, and selection of the reported results. Thus, based on the overall risk of bias from RoB 2, studies were classified into low, moderate, or high risk of bias.

### 2.6. Certainty of Evidence

Certainty of the evidence was evaluated by two independent authors (D.C.O. and R.N.C.) using the Grading of Recommendations Assessment, Development and Evaluation (GRADE) approach [34]. The GRADE is a method to assess the quality of evidence based on the risk of bias, indirectness, inconsistency, imprecision, and risk of publication bias. Similarly, any disagreements/discrepancies were resolved by a third and fourth reviewers (J.C. and L.S.M.). The criteria for one level of downgrade were (1) risk of bias of included studies: if 25% or more of the included articles had a high risk of bias as assessed by RoB 2; (2) inconsistency: if there was considerable heterogeneity (*I*^2^ > 75%); (3) indirectness: if there were differences between participants, interventions, outcome measures or indirect comparisons; (4) imprecision: if there was a wide confidence interval or its upper or lower limits spanned an effect size of 0.5, and/or small sample size (*n* < 300); and (5) risk of publication bias: if there was asymmetry in the doi plot and Luis Furuya–Kanamori (LFK) index was ±2.

### 2.7. Statistical Analyses

Statistical analysis was performed with the RStudio software (RStudio, PBC, Boston, MA) to analyze the effect of BFRT on knee extension strength (isometric and dynamic) and timed-up-and-go test (TUG). A random-effects meta-analysis was performed using the standardized mean difference (SMD) for knee extension strength and TUG, and the corresponding 95% confidence interval (CI). The heterogeneity of the studies included in the meta-analysis was evaluated through the inconsistency test (*I*^2^), represented in percentage values, interpreted using the following cutoff values: < 25% = low, 25%–50% = moderate, and > 50% = high [35]. The statistical significance of the pooled SMD was examined as Hedges' g to account for possible overestimation of the true population effect size in small studies. The estimated SMDs were interpreted as follows: SMD of 0.0–0.2 represented a trivial effect, 0.2–0.6 represented a small effect, 0.6–1.2 represented a moderate effect, 1.2–2.0 represented a large effect, 2.0–4.0 represented a very large effect, and 4.0 to represent an extremely large clinical effect [36]. A visual inspection of the DOI graph was performed to detect publication bias [37], looking for asymmetry. In addition, a quantitative measure of the LFK index was performed. An LFK index within ±1 represents no asymmetry; an LFK index greater than ±1 but within ±2 represents a minor asymmetry; and an LFK index greater than ±2 represents a major asymmetry [38]. The significance level was set at a *p* < 0.05.

To evaluate the dose-response relationship between BFRT dose and the clinical variables (knee extension strength and TUG), previously published methods were used [39]. A one-stage dose-response meta-analysis of mean differences proposed by Crippa and Orsini was performed [40, 41]. The method consisted of dose-response models estimated by pooling study-specific dose-response coefficients. Selected effect sizes and corresponding (co)variances were used to estimate study-specific dose-response curves (Supporting files 5 and 6), using the mean differences of each variable of interest and repetitions (total of repetitions during the entire BFR training program and only during the BFR exercises) as doses. This enables the characterization of the relative efficacy of the dose studied, using the placebo effect as a reference. It was used using a restricted cubic spline model with three knots at the 10th, 50th, and 90th percentiles to characterize the dose-response relationship. The procedure was implemented in the dosresmeta R package [42].

## 3. Results

### 3.1. Search Results

**Figure 1:**
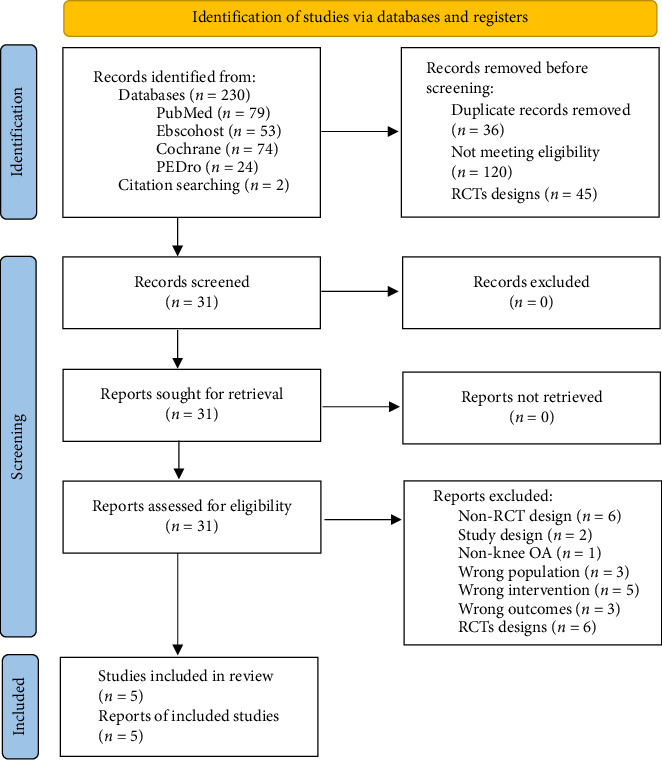
Flow diagram of the literature search and selection.

A total of 188 records were identified, and their titles and abstracts were screened. The article screening strategy is shown as a flow diagram in Figure 1. Subsequently, 31 full-text articles were assessed for eligibility, and five articles [43–47] reporting on muscle strength (knee extension) and physical function (TUG) were included in this study.

### 3.2. Assessment of Risk of Bias

**Figure 2:**
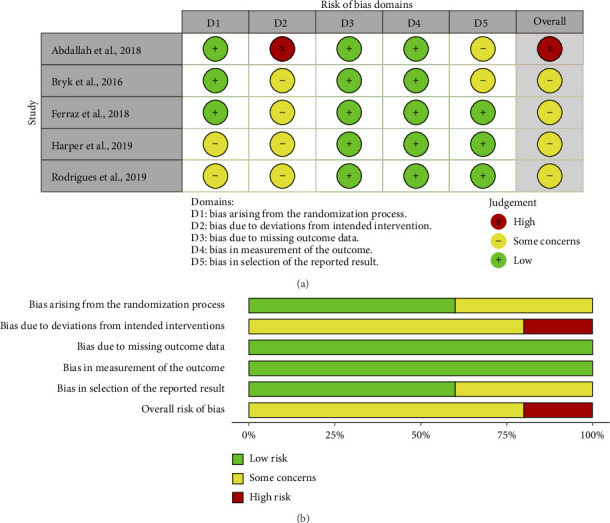
(a) Summary of the risk of bias in included studies and (b) the risk of bias as percentages across all included studies.

The risk of bias assessment of the randomized trials is shown in Figure 2. The two reviewers agreed on the scoring of 28 of the 30 final items (which include five dimensions and an overall score for each study analyzed), a 93.33% absolute agreement (*κ* = 0.884), indicating an almost perfect level of agreement. Main weaknesses of included studies were that no studies were rated as being at “low” risk. All studies had at least one domain judged as “some concerns” risk of bias. From the five studies evaluated, one [43] was judged as “high” risk of bias and four [44–47] (80%) as “some concerns”. The second domain (regarding deviations from the intended interventions) achieved the highest risk of bias in all the studies (one study with “high” [43] and four with “some concerns” [44–47]), while the third (missing outcome data) and fourth (measurement of the outcome) domains presented the lowest risk of bias with all the studies grading with “low” risk. Also, 40% of the studies exhibited “some concerns” in the randomization process [46, 47] and in the selection of the reported results [43, 44].

### 3.3. Certainty of Evidence

There is low to moderate certainty evidence in favor of BFRT for improving functionality and knee extension strength in individuals with knee OA or RA. A summary table of the results is included in Supporting File 2.

### 3.4. Study Characteristics

We included a total of 205 patients. Details of the participant's characteristics and studies are shown in Table 1. The studies compared BFRT with low loads (20%–30% 1RM) against conventional MIRT or HIRT (60%–80% 1RM). The intervention duration ranged between 4 and 12 weeks. The frequency of training ranged from 2 to 3 times/week. Further details of the BFR training protocols are shown in Table 2.

### 3.5. Outcome Measures

#### 3.5.1. Knee Extension Strength

**Figure 3:**
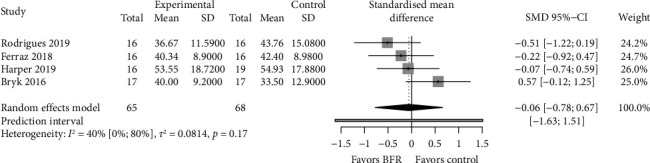
Forest plot of comparisons between BFR and control for knee extension strength.

The meta-analysis did not show statistically significant differences between interventions in 4 studies (SMD = −0.06; 95% CI = −0.78–0.67; and *p* > 0.05) without evidence of significant heterogeneity (*p*=0.15 and *I*^2^ = 40%) (Figure 3). The shape of DOI plot did not present asymmetry, and the LFK index showed no asymmetry (LFK = 0), indicating a low risk of publication bias (Supporting File 3).

#### 3.5.2. TUG

**Figure 4:**
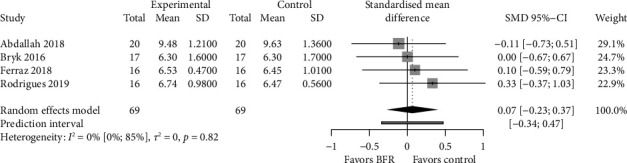
Forest plot of comparisons between BFR and control for physical function (timed up and go (TUG) test).

The meta-analysis did not show statistically significant differences between interventions in 4 studies (SMD = 0.07.; 95% CI = −0.23–0.37; and *p* > 0.05) without evidence of significant heterogeneity (*p*=0.82 and *I*^2^ = 0%) (Figure 4). The shape of DOI plot presented asymmetry, and the LFK index showed minor asymmetry (LFK = 1.19), indicating a risk of publication bias (Supporting File 4).

### 3.6. Dose Response

#### 3.6.1. Knee Extension Strength

**Figure 5:**
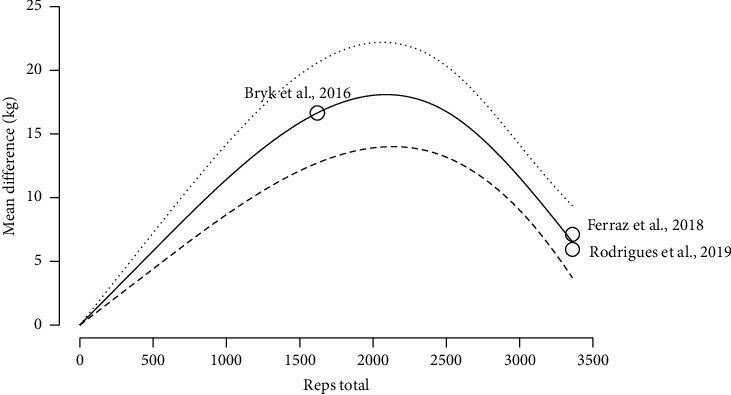
Knee extension strength BFR training dose-response.

The report by Harper et al. [46] was excluded from the dose-response analysis because their participants went to volitional failure, while the other studies were structured around a fixed repetition scheme. The results indicated a statistically significant negative association between increasing doses of BFRT (total repetitions) and the mean change in knee extension strength, with the maximum value of 18.03 (95% CI: 13.90, 22.15) observed at dose max = 2000 reps total (Figure 5 and Supporting File 7).

#### 3.6.2. TUG

**Figure 6:**
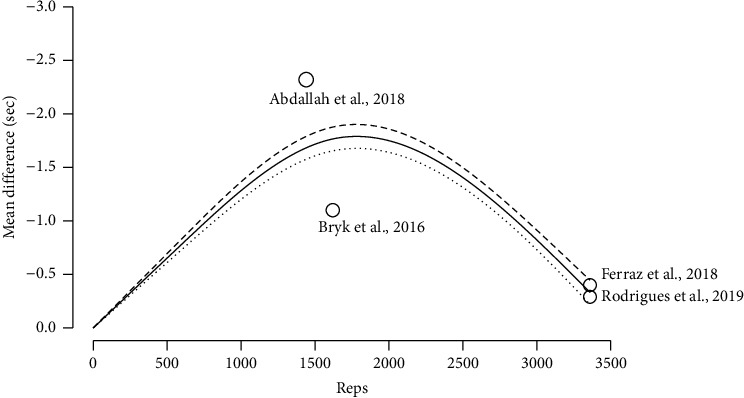
TUG BFR training dose-response.

The results indicated a statistically significant negative association between increasing doses of BFRT (total repetitions) and the mean change in TUG score with the maximum value of −1.36 (95% CI: 0.96, 1.76) observed at dose max = 1800 reps total (Figure 6 and Supporting File 8).

## 4. Discussion

This systematic review with dose-response meta-analysis was performed to analyze the effects of BFRT on strength and physical function in the treatment of knee OA or RA. The main findings were that (1) BFRT resulted in significant improvements in strength and functionality in individuals with knee OA or RA similar to conventional MIRT and HIRT; (2) a total of 2000 repetitions per BFRT program are required to maximize strength gains in these individuals; and that (3) maximal improvement of physical function was observed with approximately 1800 total repetitions.

The pooled data of the included studies did not show significant differences between BFRT and MIRT or HIRT in individuals with knee OA or RA for knee extension strength and physical function (TUG). Previous meta-analyses have shown that BFRT produces similar effects to MIRT and HIRT in improving strength and functionality in subjects with knee RA [23] and OA [23–28]. Dos Santos et al. [23] highlight that MIRT and HIRT are extremely effective in producing gains in strength and functionality, so the findings for BFRT are highly important and promising for these populations. Similarly, other systematic reviews also report similar results between BFRT and HIRT in strength and functionality gains in subjects with knee OA [26–48] or in patients after anterior cruciate ligament reconstruction [48]. However, Pitsillides et al. [26] report that HIRT protocols may have been applied improperly due to the pain produced, as the 1RM may have been the maximum load tolerated instead of the real maximum load, so the similar strength values in BFRT and HIRT groups could have been altered for this reason. The meta-analysis conducted by Grantham et al. [27], found similar results in strength and objective physical function (and no difference for the other clinical outcomes (pain, self-reported function, and muscle size)) between BFRT and HIRT in individuals with knee OA. Besides effectiveness, a previous systematic review found BFRT safe when used according to evidence-based guidelines in patients with various knee-related musculoskeletal disorders [49]. That finding is further backed by Wang et al. in their more recent meta-analysis that showed BFRT has less risk of adverse effects than HIRT [28]. Research thus far is promising with respect to safety and efficacy outcomes in a variety of elderly and clinical populations, with minimal incidence of adverse effects [50–58]. Of the included studies [43–47], only two reported adverse events in the BFRT group, with most of these events being related to knee pain (or mild discomfort regarding occlusion pressure in the first sessions) [46, 47]. However, in aggregate, the BFRT group had less of these reports than traditional MIRT or HIRT. Importantly, the fear of experiencing discomfort or exacerbating existing pain during high-intensity strength training can be a major barrier to exercise adherence in patients with knee OA or RA, an issue that is significantly reduced with BFRT due to its low-load nature. Thus, beyond the comparison of efficacy and safety, the critical question might be which of the interventions—BFRT, MIRT, or HIRT—provides the greatest balance of benefits with minimal risk and discomfort, thus promoting the highest level of patient adherence and long-term commitment to an exercise program. While some aspects of BFRT are often considered as limitations to its implementation and clinical relevance, like lack of equipment and insufficient training [59], commonly, any kind of training modality requires some kind of equipment and some level of training. Nonetheless, low-cost approaches that could help make BFRT safer and more accessible to clinicians, and even for home use, are becoming more readily available [60–62]. Using pneumatic cuffs designed for BFRT, or even elastic wraps as an even more economic equivalent, and procedures that obviate the need for individualized objective measurement of limb occlusion pressures, allows for BFRT implementations that are safe and do not require costly equipment [60–62].

The major novelty of this study is the estimation, through a dose-response meta-analysis, of the total training volume required to maximize the improvements in strength and functionality with BFRT for patients with knee OA and RA. Interestingly, the BFRT doses differ depending on the outcome of interest. Specifically, we observed that the curve describing the relationship between the increase in repetitions in a BFRT program and knee extension strength has an inverted U-shape. According to our results, 2000 total repetitions are necessary to achieve maximal strength improvement. As an example, following the standard 75 rep scheme, at a frequency of 3 sessions per week, that would amount to about 9 weeks of BFRT, which would be in line with Pitsillides's et al. and dos Santos's et al. recommendations [23–26]. However, another possible practical application would be to perform two exercises per muscle group, potentially halving the required time. In addition, a training frequency of at least 3 times per week also seems to be supported in young and older adults [50–63]. For physical function, we observed that the relationship between BFRT repetitions and TUG also has an inverted U-shape, where the greatest improvements in functionality are found at 1800 total repetitions. Similarly to strength, if following the standard 75 rep scheme, at a frequency of 3 sessions per week, that would amount to about 7 weeks of BFRT. From a strength perspective, as the model suggested a decrease in the predicted strength gains for doses above 2000 total repetitions, our results would imply that there is a certain threshold of volume that can be used in a BFRT program, over which further increases in volume are not advantageous. It might be that higher BFRT volumes over longer time courses limit the breadth of muscle-related adaptations to resistance training in this population. That might be because of a different time course of metabolic signaling or neurophysiological adaptations with BFRT, different responses based on training status, the lack of periodization due to the intervention designs, the attenuated muscle anabolic response with age or pathology, or delayed or impaired recovery from exercise. A recent meta-analysis showed that responses to BFRT regarding the magnitudes of muscle strength and hypertrophy adaptations do indeed differ based on individual training status, being greater for trained than untrained individuals, compared with HIRT [64]. It is also conceivable that the dose-response relationship may be moderated by the implemented intervention designs, which ranged from 4 to 12 weeks. As the included studies made use of no periodization model, our analysis of the response patterns suggests that further intensity progression may be necessary during the training program to sustain adaptive responses. Given that the exact physiological mechanisms underlying BFRT are still hotly debated and that most of the research on periodization has been conducted on other training modalities and in healthy individuals, we can only speculate on the causes of the inverted U-shape of the dose-response relationship. Exploring this topic in future studies using longer duration interventions and additional outcome measures might further the understanding of our findings. Similarly, this decrease in physical function at higher training volumes may be because improvements in functionality are not only influenced by strength but also require improving coordination, proprioception, and mobility. Therefore, even though strength is very important to improve functionality, other factors should not be ignored if wanting to optimize functionality.

To our knowledge this is the first dose-response meta-analysis that investigates the effects of BFRT on lower limb strength and functionality in individuals with knee OA or RA. Our findings may have important clinical implications for the treatment of these patient populations in the future.

### 4.1. Limitations

The limitations to our research are those inherent to this type of quantitative analysis, such as the constraints imposed by the low number, small sample sizes, and methodological heterogeneity of the studies published to date in these populations as well as publication bias, which might bias the results obtained in the meta-analysis. As such, it is important to recognize that, due to the small sample sizes, our results represent trends in strength gains and physical function improvements and cannot be considered a definitive recommendation. Only improvements in muscle strength were considered, as we could not analyze muscle hypertrophy due to these constraints. In the same vein, we pooled together dynamic and isometric maximal strength measures to allow for meta-analysis. Similarly, our analysis of physical function was constrained to the TUG test. Finally, the risk of bias in the included studies was mainly affected by the domain of “deviations from planned interventions”, which highlights the need for researchers to publish a priori complete protocols and write their reports with more details. Despite these limitations, our findings represent an important advance in understanding the role of BFRT in treating muscle weakness and functionality loss in individuals with knee OA or RA. Further high-quality, large-scale, randomized controlled trials that investigate the effects of BFRT on myoelectric activity, muscle hypertrophy, pain, diverse functionality tests, safety and long-term adherence in these populations are necessary to confirm and extend our findings. Moreover, standardized reporting of methodology and results, including complete intervention protocols, should be prioritized in future research to enhance transparency and reproducibility. Future randomized controlled trials should corroborate our findings and evaluate the effects of different BFRT dosing and to address the evidence gap around the sustainability of outcome measures with programs that are longer than 12 weeks as to ascertain whether the positive effects persist beyond the initial study period or they are likely to fade over time.

## 5. Conclusions

This dose-response meta-analysis identifies significant improvements in strength and functionality after performing BFRT in individuals with knee OA or RA similar to conventional moderate-high intensity resistance training. A total of 2000 repetitions per BFRT program are necessary to maximize strength gains in these patients, while maximal functional improvement requires 1800 total repetitions. Following a one exercise standard 75 rep BFRT scheme, at a frequency of 3 sessions per week, about 9 weeks of BFRT for knee extension strength, and 7 weeks for physical function would maximize the gains achievable through this modality. BFRT is a promising intervention, especially for patients who cannot tolerate high training load, and could be an important therapeutic strategy to maintain and improve their strength and functionality.

## Figures and Tables

**Table 1:** Analysis of included studies.

Author, year [references]	Sample size, age, BMI	Population	Sex	Intervention	Control/comparator	Outcome measures	Summary of findings	Adverse effects
Abdallah et al., 2021 [41]	*n* = 40 48.55/48.85 years BMI = 26.25/26.21	KOA	Male	Progressive LLRT with BFR	Progressive HLRT	TUG test	There were significant improvements in TUG test between pre- and postintervention in both groups (*p*=0.0001; % of change: 20.3 for BFRT vs. 17.4 HLRT), but no significant difference between groups either before (*p*=0.311) or after treatment (*p*=0.358).	No adverse effects were reported.
Bryk et al., 2016 [42]	*n* = 34 60.4/62.3 years BMI = 30.8/28.9	KOA	Female	Progressive LLRT with BFR	Progressive HLRT	Quadriceps MVIC and TUG test	In both groups, a significant difference was found compared with the baseline in quadriceps MVIC (*p*=0.001; mean ± SD change score: 16.8 ± 10.3 for BFRT vs. 9.4 ± 8.3 HLRT) and TUG (*p*=0.006; mean ± SD change score: −1.2 ± 1.8 for BFRT vs. −1.6 ± 3.5 HLRT), but no significant differences were found between groups.	No adverse effects were reported.
Ferraz et al., 2018 [43]	*n* = 48 59.9/60.7/60.3 years BMI = 30.3/29.9/30.2	KOA	Female	Progressive LLRT with BFR	Progressive HLRT and progressive LLRT	Leg press 1RM, knee extension 1RM, and TUG test	HLRT and BFRT had significant improvements in leg press 1RM (33% vs. 26%; *p* < 0.0001 and *p*=0.0004) and knee extension 1RM (22% vs. 23%; *p*=0.0004 and *p*=0.0005) compared with LLRT. There were no significant differences between HLRT and LLRT with BFR (*p* > 0.05). No intragroup (*p* > 0.05) or between-group improvements in the TUG (*p* > 0.05).	Four patients from the HLRT group were excluded during follow-up due to exercise-induced knee pain.
Harper et al., 2019 [44]	*n* = 35 69.1/67.2 years BMI = 29.8/31.7	KOA	Male and female	Progressive BFR	Progressive MIRT	Isokinetic knee extensors strength (dynamometer)	Both groups showed an improvement in strength, with a pre- to posttraining change in the mean composite knee extensor peak torque of 9.96 (5.76, 14.16) Nm. The mean change between groups for mean composite knee extensor peak torque at Week 12 was (BFR relative to MIRT) −1.87 (−10.96, 7.23) Nm.	Seven adverse events (excluding expected reports of knee pain) related or possibly related to the study were reported.
Rodrigues et al., 2019 [45]	*n* = 48 68/59.6/58.1 years BMI = 24.7/27.4/26.9	RA	Female	Progressive BFRT	Progressive HLRT and No exercise control.	Leg press 1RM, knee extension 1RM, and TUG test	HLRT and BFRT had significant improvements in leg press 1RM (% of change: 24.2% vs. 22.8%; *p* < 0.0001 and *p* < 0.0001) and knee extension 1RM (% of change: 23.8% vs. 19.7%; *p* < 0.0001 and *p* < 0.0001) compared with control. There were no significant differences between HLRT and BFRT (*p* > 0.05), indicating a similar effect between treatments. HLRT and BFRT had significant improvements in TUG test (% of change: −8.7% vs. −6.8%; *p* < 0.0001 and *p*=0.0053). There were no significant differences between HLRT and BFRT (*p* > 0.05), indicating a similar effect between treatments.	One participant (HLRT) dropped out due to exercise-induced patellofemoral pain. Another 8 (HLRT) reported knee pain, requiring load/reps reduction up to 2 sessions. In the BFRT group, 6 patients reported transitory discomfort due to occlusion in the first sessions, but load adjustments were not necessary.

Abbreviations: BFR, blood flow restriction; BFRT, blood flow restriction training; BMI, body mass index; HLRT, high-load resistance training; KOA, knee osteoarthritis; LLRT, low-load resistance training; MIRT, moderate intensity resistance training; MVIC, maximum voluntary isometric contraction; RA, rheumatoid arthritis; RM, repetition maximum; TUG, timed up-and-go test.

**Table 2:** Details of the BFR training protocols.

Author, year [references]	BFR intervention				Control/comparator		
	Modality and exercises	Cuff (size, placement, pressure)	Intensity and volume	Duration and frequency	Modality and exercises	Intensity and volume	Duration and frequency
Abdallah et al., 2021 [41]	Traditional BFR Constant BFR • Straight leg raises (in supine). • Knee extension (in seated position). • Hip abduction and adduction (in lateral decubitus). • Unilateral calf raises. • Hamstring stretch (in supine).	Size = 5–6 cm width Placement = 33% distal to the inguinal crease. Pressure = 200 mmHg	30% 1RM 10 reps × 3 sets 30 s × 3 sets (hamstring stretch)	4 weeks 3 times/week	HLRT • Straight leg raises (in supine). • Knee extension (in seated position). • Hip abduction and adduction (in lateral decubitus). • Unilateral calf raises. • Hamstring stretch (in supine).	60% 1RM 10 reps × 3 sets 30 s × 3 sets (hamstring stretch)	4 weeks 3 times/week
Bryk et al., 2016 [42]	Traditional BFR Constant BFR • Hamstring stretches. • Glute bridge with isometric transversus abdominis contraction (core training). • Hip abductions (in side-lying position). • Hip adductions (in side-lying position). • Calf raises. • Clam exercise (in side-lying position) with a band that allows for a 10RM. • Sensorimotor training (standing). • Knee extension in seated position with BFR.	Size = not specified Placement = upper third of the thigh Pressure = 200 mmHg	Exercises in common: 70% 1RM. Exercises with BFR: 30% 1RM. 10 reps × 3 sets 30 reps × 3 sets (BFR) 30 s × 3 sets (stretches, sensorimotor)	6 weeks 3 times/week	HLRT • Hamstring stretches. • Glute bridge with isometric transversus abdominis contraction (core training). • Hip abductions (in side-lying position). • Hip adductions (in side-lying position). • Calf raises. • Clam exercise (in side-lying position) with a band that allows for a 10RM. • Sensorimotor training (standing). • Knee extension in seated position.	70% 1RM 10 reps × 3 sets 30 s × 3 sets (stretches, sensorimotor)	6 weeks 3 times/week
Ferraz et al., 2018 [43]	Traditional BFR Constant BFR • Leg press • Knee extension	Size = 175 mm × 920 mm Placement = Inguinal fold Pressure = 70% AOP	20% 1RM first week. 30% 1RM from the second week. 15 reps × 4 sets (week 1–4) 15 reps × 5 sets (week 5–12)	12 weeks 2 times/week	HLRT and LLRT • Leg press • Knee extension	HLRT 50% 1RM first week. 80% 1RM from the second week. 10 reps × 4 sets (week 1–4) 10 reps × 5 sets (week 5–12) LLRT 20% 1RM first week. 30% 1RM from the second week. 15 reps × 4 sets (week 1–4) 15 reps × 5 sets (week 5–12)	12 weeks 2 times/week
Harper et al., 2019 [44]	Traditional BFR Constant BFR • Leg press • Knee extension • Knee flexion • Calf raises	Size = not specified. Placement = proximal part of the thigh Pressure = 0.5 × (SBP) + 2 × (thigh circumference) + 5	20% 1RM Exercises performed to voluntary fatigue.	12 weeks 3 times/week	MIRT • Leg press • Knee extension • Knee flexion • Calf raises	60% 1RM Exercises performed to voluntary fatigue.	12 weeks 3 times/week
Rodrigues et al., 2019 [45]	Traditional Constant • Leg press • Knee extension	Size = 175 mm × 920 mm Placement = Inguinal fold Pressure = 70% AOP	20% 1RM first week. 30% 1RM from the second week. 15 reps × 4 sets (week 1–4) 15 reps × 5 sets (week 5–12)	12 weeks 2 times/week	HLRT and control group. HLRT: • Leg press • Knee extension Control group: • No exercise.	HLRT 50% 1RM first week. 70% 1RM from the second week. 10 reps × 4 sets (week 1–4) 10 reps × 5 sets (week 5–12) Control No exercise.	12 weeks 2 times/week

Abbreviations: AOP, arterial occlusion pressure; BFR, blood flow restriction; HLRT, high-load resistance training; LLRT, low-load resistance training; MIRT, moderate intensity resistance training; RM, repetition maximum; SBP, systolic blood pressure.

## Data Availability

The literature data supporting this publication are from previously reported studies and datasets, which have been cited. The processed data are available in Tables 1 and 2 and Figures 1, 2, 3, 4, 5, and 6, the Supporting Information of this article, and from the corresponding author upon request.

## Supporting Information

Supporting Information 1 Supporting File 1: Keywords Strategy.

Supporting Information 2 Supporting File 2: Assessment of the quality of evidence through the GRADE system.

Supporting Information 3 Supporting File 3: Strength doi plot.

Supporting Information 4 Supporting File 4: TUG doi plot.

Supporting Information 5 Supporting File 5: Selected effect sizes and data used to estimate strength dose-response meta-analysis.

Supporting Information 6 Supporting File 6: Selected effect sizes and data used to estimate TUG dose-response meta-analysis.

Supporting Information 7 Supporting File 7: Results of dose-response (dose and predicted mean difference) of strength variable.

Supporting Information 8 Supporting File 8: Results of dose-response (dose and predicted mean difference) of TUG variable.
