# Supplementary material for: Effects of Blood Flow Restriction Training on Strength and Functionality in People With Knee Arthropathies: A Systematic Review and Dose-Response Meta-Analysis of Randomized Controlled Trials
Source: Transl Sports Med. 2025 Apr 10;2025:3663009. doi: 10.1155/tsm2/3663009 (PMC12006712; doi:10.1155/tsm2/3663009)
Supplement: Supporting Information 1 — Supporting File 1: Keywords Strategy. [file 3663009.f1.docx]

**Supplemental File 1: Keywords Strategy**

**PubMed**

Query String:

("blood flow restriction therapy"[Mesh] OR (blood[all] AND flow[all] AND restriction[all] AND therapy[all]) OR "blood flow restriction therapy"[all] OR (blood[all] AND flow[all] AND restriction[all] AND training[all]) OR "blood flow restriction training"[all] OR ("blood flow restriction therapy"[Mesh] OR (blood[all] AND flow[all] AND restriction[all] AND therapy[all]) OR (blood[all] AND flow[all] AND restriction[all] AND exercise[all]) OR "blood flow restriction exercise"[all]) OR KAATSU[all]) AND (Osteoarthritis OR "rheumatoid arthritis")

Results of preliminary search (19 feb 2023): 68.

Results of definitive search (29 jun 2024): 79.

**Ebsco**

Query String:

((MH "blood flow restriction therapy+") OR (blood AND flow AND restriction AND therapy) OR "blood flow restriction therapy" OR (blood AND flow AND restriction AND training) OR "blood flow restriction training" OR ((MH "blood flow restriction therapy+") OR (blood AND flow AND restriction AND therapy) OR "blood flow restriction therapy" OR (blood AND flow AND restriction AND exercise) OR "blood flow restriction exercise") OR ((MH "blood flow restriction therapy+") OR (blood AND flow AND restriction AND therapy) OR "blood flow restriction therapy") OR KAATSU) AND (Osteoarthritis OR "rheumatoid arthritis")

Results of preliminary search (19 feb 2023): 43.

Results of definitive search (29 jun 2024): 53.

**Cochrane Library**

Query String:

("blood flow restriction" OR KAATSU) AND (Osteoarthritis OR "rheumatoid arthritis")

Results of preliminary search (19 feb 2023): 58.

Results of definitive search (29 jun 2024): 74.

**PEDro**

Query String 1:

"blood flow restriction" AND Osteoarthritis

Results of preliminary search (19 feb 2023): 16.

Results of definitive search (29 jun 2024): 21.

Query String 2:

"blood flow restriction" AND "rheumatoid arthritis"

Results of preliminary search (19 feb 2023): 3.

Results of definitive search (29 jun 2024): 3.
