# Supplementary material for: Effects of Blood Flow Restriction Training on Strength and Functionality in People With Knee Arthropathies: A Systematic Review and Dose-Response Meta-Analysis of Randomized Controlled Trials
Source: Transl Sports Med. 2025 Apr 10;2025:3663009. doi: 10.1155/tsm2/3663009 (PMC12006712; doi:10.1155/tsm2/3663009)
Supplement: Supporting Information 2 — Supporting File 2: Assessment of the quality of evidence through the GRADE system. [file 3663009.f2.docx]

**Supplemental File X. Assessment of the quality of evidence through the GRADE system**

Summary of findings for the effects of BFRT on strength and functionality compared to conventional RT

| Certainty Assessment | | | | | | | Effect:  SMD (95% CI) | Certainty |
| --- | --- | --- | --- | --- | --- | --- | --- | --- |
| No. of Studies | Study Design | Risk of Bias | Inconsistency | Indirectness | Imprecision | Other Considerations |  |  |
| Strength | | | | | | | | |
| 4 | RCT | Not serious | Not serious | Not serious | Serious^a^ | None | -0.11 (-0.86 to 0.63) | ⨁⨁⨁◯  MODERATE |
| Functionality | | | | | | | | |
| 4 | RCT | Not serious | Not serious | Not serious | Serious^a,b^ | Publication bias^c^ | 0.07 (-0.23 to 0.37) | ⨁⨁◯◯  LOW |

RCT: Randomized Controlled Trial; CI: Confidence interval; SMD: Standardized mean difference; LFK: Luis Furuya-Kanamori.

^a^ <300 participants combined for each outcome (serious imprecision).

^b^ upper or lower CI spanned an effect size of 0.5 (serious imprecision).

^c^ The shape of DOI plot presented asymmetry, and the LFK index showed minor asymmetry (LFK= 1.14) indicating a risk of publication bias.

*Quality of evidence:*

High: We are very confident that the true effect lies close to that of the estimate of the effect.

Moderate: We are moderately confident in the effect estimate: the true effect is likely to be close to the estimate of the effect, but there is a possibility that it is substantially different.

Low: Our confidence in the effect estimate is limited: the true effect may be substantially different from the estimate of the effect.

Very low: We have very little confidence in the effect estimate: the true effect is likely to be substantially different from the estimate of the effect.
