# Supplementary figures and images for: Effects of Blood Flow Restriction Training on Strength and Functionality in People With Knee Arthropathies: A Systematic Review and Dose-Response Meta-Analysis of Randomized Controlled Trials
Source: Transl Sports Med. 2025 Apr 10;2025:3663009. doi: 10.1155/tsm2/3663009 (PMC12006712; doi:10.1155/tsm2/3663009)

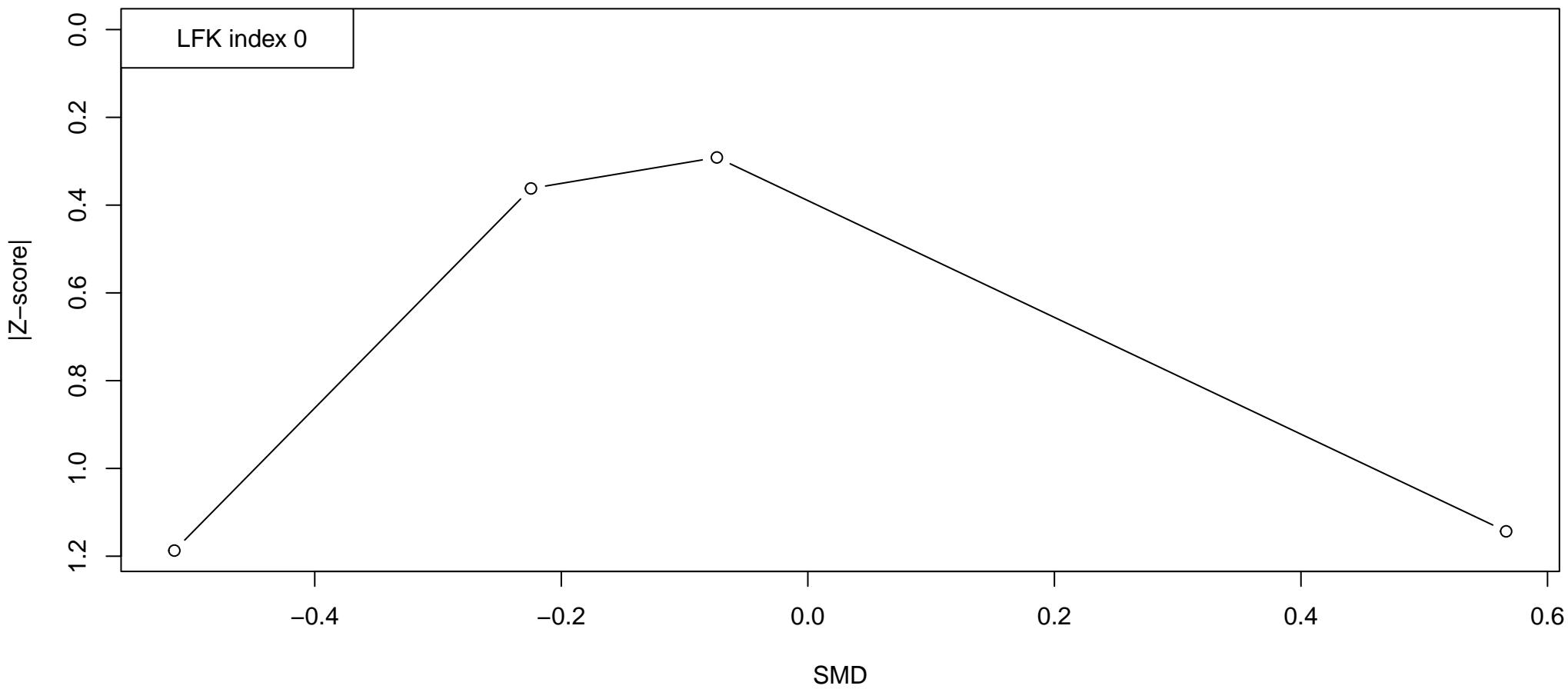

Supplement: Supporting Information 3 — Supporting File 3: Strength doi plot. [file 3663009.f3.pdf]

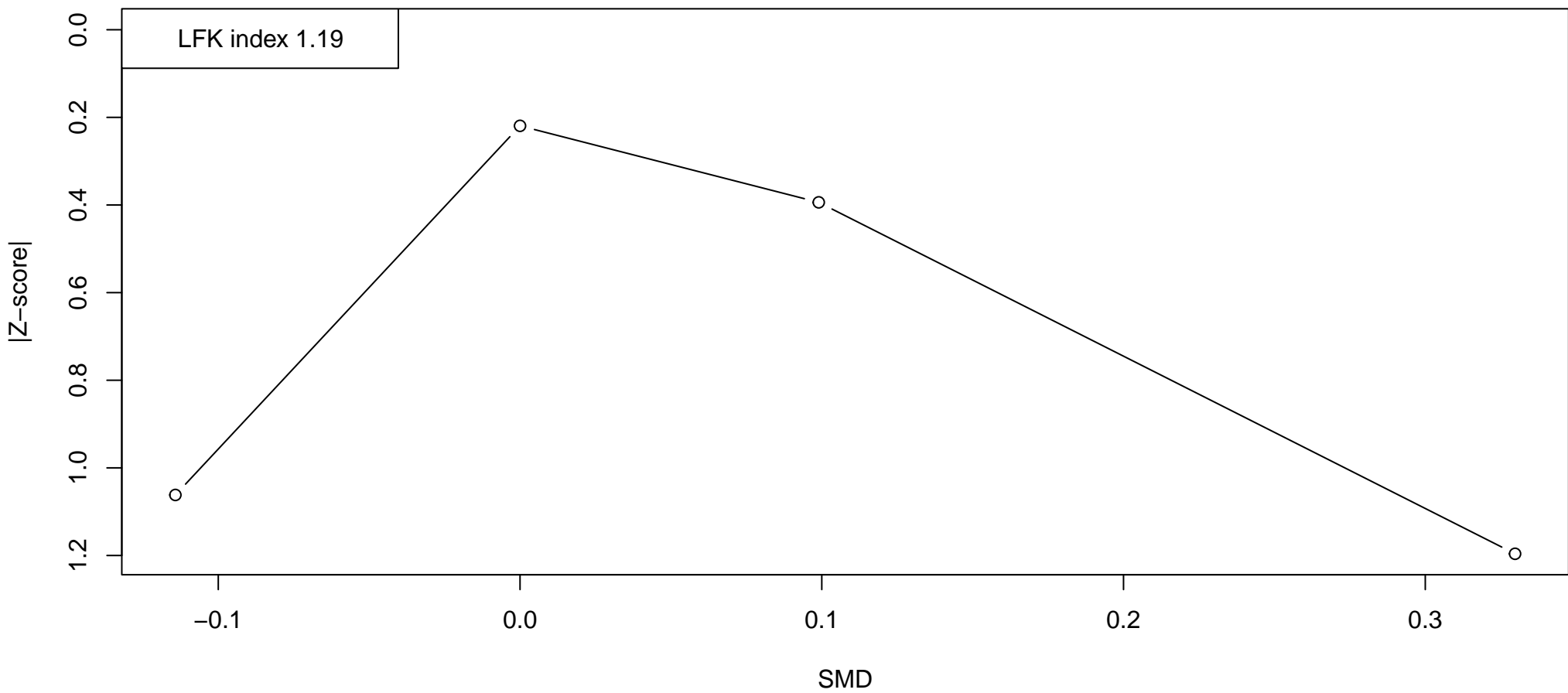

Supplement: Supporting Information 4 — Supporting File 4: TUG doi plot. [file 3663009.f4.pdf]
