# Supplementary material for: Effects of Blood Flow Restriction Training on Strength and Functionality in People With Knee Arthropathies: A Systematic Review and Dose-Response Meta-Analysis of Randomized Controlled Trials
Source: Transl Sports Med. 2025 Apr 10;2025:3663009. doi: 10.1155/tsm2/3663009 (PMC12006712; doi:10.1155/tsm2/3663009)
Supplement: Supporting Information 6 — Supporting File 6: Selected effect sizes and data used to estimate TUG dose-response meta-analysis. [file 3663009.f6.docx]

| **author** | **dose** | **y** | **sd** | **n** |
| --- | --- | --- | --- | --- |
| Ferraz 2018 (control group) | 0 | 0,02 | 0,05 | 16 |
| Ferraz 2018 | 3360 | -0,37 | 0,2 | 16 |
| Bryk 2016 | 1620 | -1,2 | 1,8 | 17 |
| Rodrigues 2019 | 3360 | -0,5 | 0,3 | 16 |
| Abdallah 2018 | 1440 | -2,4 | 1,21 | 20 |

**Supplemental file 6.** Selected effect sizes and data used to estimate TUG dose-response meta-analysis.
