# Supplementary material for: Effects of Blood Flow Restriction Training on Strength and Functionality in People With Knee Arthropathies: A Systematic Review and Dose-Response Meta-Analysis of Randomized Controlled Trials
Source: Transl Sports Med. 2025 Apr 10;2025:3663009. doi: 10.1155/tsm2/3663009 (PMC12006712; doi:10.1155/tsm2/3663009)
Supplement: Supporting Information 7 — Supporting File 7: Results of dose-response (dose and predicted mean difference) of strength variable. [file 3663009.f7.docx]

**Supplemental file 7.** Results of dose-response (dose and predicted mean difference) of strength variable.

| **Dose** | **Pred** | **ci.lb** | **ci.ub** |
| --- | --- | --- | --- |
| 0 | 0.000000 | 0.000000 | 0.000000 |
| 200 | 2.327499 | 1.762791 | 2.892206 |
| 400 | 4.654997 | 3.525582 | 5.784413 |
| 600 | 6.980258 | 5.286796 | 8.673720 |
| 800 | 9.263230 | 7.018213 | 11.508248 |
| 1000 | 11.432371 | 8.669380 | 14.195361 |
| 1200 | 13.415174 | 10.189025 | 16.641323 |
| 1400 | 15.139137 | 11.525574 | 18.752700 |
| 1600 | 16.531754 | 12.626901 | 20.436607 |
| 1800 | 17.520521 | 13.439903 | 21.601138 |
| 2000 | 18.032933 | 13.909676 | 22.156189 |
| 2200 | 17.996486 | 13.977785 | 22.015187 |
| 2400 | 17.338675 | 13.578275 | 21.099076 |
| 2600 | 15.993638 | 12.632408 | 19.354868 |
| 2800 | 14.017599 | 11.113574 | 16.921624 |
| 3000 | 11.573931 | 9.014236 | 14.133626 |
| 3200 | 8.829644 | 6.291114 | 11.368174 |
| 3400 | 5.951526 | 3.026379 | 8.876673 |
