# Supplementary material for: Effects of Blood Flow Restriction Training on Strength and Functionality in People With Knee Arthropathies: A Systematic Review and Dose-Response Meta-Analysis of Randomized Controlled Trials
Source: Transl Sports Med. 2025 Apr 10;2025:3663009. doi: 10.1155/tsm2/3663009 (PMC12006712; doi:10.1155/tsm2/3663009)
Supplement: Supporting Information 8 — Supporting File 8: Results of dose-response (dose and predicted mean difference) of TUG variable. [file 3663009.f8.docx]

**Supplemental file 8.** Results of dose-response (dose and predicted mean difference) of TUG variable.

| **Dose** | **Pred** | **ci.lb** | **ci.ub** |
| --- | --- | --- | --- |
| 0 | 0.0000000 | 0.00000000 | 0.0000000 |
| 200 | 0.2129023 | 0.14750810 | 0.2782964 |
| 400 | 0.4258045 | 0.29501620 | 0.5565929 |
| 600 | 0.6387024 | 0.44252160 | 0.8348832 |
| 800 | 0.8480255 | 0.58783006 | 1.1082209 |
| 1000 | 1.0402077 | 0.72256642 | 1.3578489 |
| 1200 | 1.1999446 | 0.83705424 | 1.5628349 |
| 1400 | 1.3119318 | 0.92096192 | 1.7029018 |
| 1600 | 1.3630929 | 0.96388697 | 1.7622989 |
| 1800 | 1.3868164 | 0.96374163 | 1.7498913 |
| 2000 | 1.3038706 | 0.92116028 | 1.6865809 |
| 2200 | 1.2150585 | 0.83545856 | 1.5946585 |
| 2400 | 1.1011833 | 0.70730838 | 1.4950582 |
| 2600 | 0.9730480 | 0.54302757 | 1.4030684 |
| 2800 | 0.8408895 | 0.35551937 | 1.3262597 |
| 3000 | 0.7086721 | 0.15435434 | 1.2629898 |
| 3200 | 0.5764546 | -0.05597411 | 1.2088834 |
| 3400 | 0.4442372 | -0.27247620 | 1.1609506 |
